# Trends in perception of generative AI in healthcare by US adult demographics and political identity

**DOI:** 10.1093/haschl/qxag148

**Published:** 2026-06-10

**Authors:** Benjamin Rader, Autumn H Gertz, Jonathan H Cantor, Ryan K McBain, John S Brownstein

**Affiliations:** Boston Children's Hospital, 300 Longwood Ave, Boston, MA 02115, United States; Harvard Medical School, Boston, MA 02115, United States; Boston Children's Hospital, 300 Longwood Ave, Boston, MA 02115, United States; RAND, Santa Monica, CA 90401, United States; Harvard Medical School, Boston, MA 02115, United States; RAND, Arlington, VA 22202, United States; Mass General Brigham, Boston, MA 02115, United States; Boston Children's Hospital, 300 Longwood Ave, Boston, MA 02115, United States; Harvard Medical School, Boston, MA 02115, United States

**Keywords:** public opinion, trust, generative AI, health equity, partisan divide, digital health

## Abstract

**Introduction:**

Generative AI (genAI) is rapidly entering healthcare, but public comfort may vary in ways that could undermine equitable adoption.

**Methods:**

Using two independent cross-sectional survey waves of US adults conducted in May 2023 (*N* = 3130) and November 2025 (*N* = 3088) on the Outbreaks Near Me participatory surveillance platform, we assessed changes in awareness of genAI and four comfort and perception measures.

**Results:**

The percentage of respondents reporting they had heard “a lot” about genAI rose from 26.5% (95% confidence interval [CI]: 24.5, 28.5) to 42.9% (95% CI: 40.6, 45.3), yet overall comfort with AI-led primary care and AI-led therapy changed minimally (−1.0 and −0.7% points, respectively). Subgroup trends diverged sharply by political identity, age, and race. Notably, Republicans trended more favorably from 2023 to 2025 while Democrats declined (interaction odds ratios across the four measures ranging from 1.69 [95% CI: 1.19, 2.39] to 2.11 [95% CI: 1.54, 2.90]).

**Conclusion:**

Emerging divides suggest receptivity to genAI in healthcare may become increasingly shaped by political identity rather than clinical evidence or personal experience, complicating equitable deployment.

Key points

- Awareness of generative AI nearly doubled between 2023 and 2025, but comfort with AI-led care did not move. Successful long-term deployment of AI in healthcare will require more than just exposure via implementation.
- Attitudes toward AI in healthcare are splitting along partisan lines. Equitable AI deployment will depend on reaching the populations growing most resistant.

## Introduction

The landscape surrounding generative AI (genAI) in healthcare has shifted considerably in recent years.^1-3^ In 2025, three in five US adults reported using AI tools for health-related purposes,^4^ even as safety concerns and state-level legislation regulating AI in healthcare have heightened public skepticism.^5-8^ Cross-sectional surveys have documented variation in comfort with genAI in healthcare by age, political leaning, and education.^9-11^ Whether public sentiment has shifted alongside these rapid genAI developments, and whether demographic differences in comfort with AI are widening, narrowing, or remaining stable, remains an open question.^11^

In May 2023, following the public release of ChatGPT, we fielded a census-weighted national participatory surveillance survey to understand sentiment on genAI in healthcare. In November 2025, we repeated the survey. Here, we compare these periods, focusing on changes by demographic characteristics and political identity. As the AI landscape continues to evolve, understanding how attitudes shift is essential to informing policies that promote equitable genAI integration.

## Data and methods

In two independent cross-sectional waves (May 23–26, 2023 and November 4–6, 2025), we queried US adults (≥18) on their self-reported demographic characteristics, political leanings, occupation and their awareness of, perceptions of, and comfort with genAI in healthcare.

Data were collected through Outbreaks Near Me (ONM), a web-based participatory surveillance platform that recruits users nationwide to self-report health information and related attitudes.^12^ ONM leverages an end-page river sampling strategy on SurveyMonkey. When a SurveyMonkey user finishes an unrelated survey, they are invited to participate in ONM, enabling rapid recruitment of a heterogeneous sample. ONM has demonstrated strong alignment with traditional public health surveillance, including national at-home COVID-19 test use and CDC estimates of chronic illness.^12-14^

We descriptively compared the percentage endorsing each answer across waves and by demographic and political subgroup. To statistically test whether changes between waves differed by subgroup, we estimated multivariable logistic regression models for each outcome with survey year, demographic variable, and their interaction, and report the exponentiated interaction terms (ORs). To assess whether healthcare worker status modified the partisan divergence, we first fit exploratory models including a survey year-by-political identity-by-healthcare worker status interaction. We then evaluated whether geography confounded the partisan differences by adjusting for urban, suburban, or rural residence, defined using Rural-Urban Commuting Area codes. All analyses used survey weights calibrated to match US Census demographics, including gender, education, race, ethnicity, income, age, and geography. Respondents missing any of these weighting variables were excluded from the sample. Weighted and unweighted demographics are compared to US Census population estimates in Table S1. Analyses were performed in R (v4.3.2). This study was deemed exempt by the BCH IRB (P00023700).

## Results

**Figure 1:**
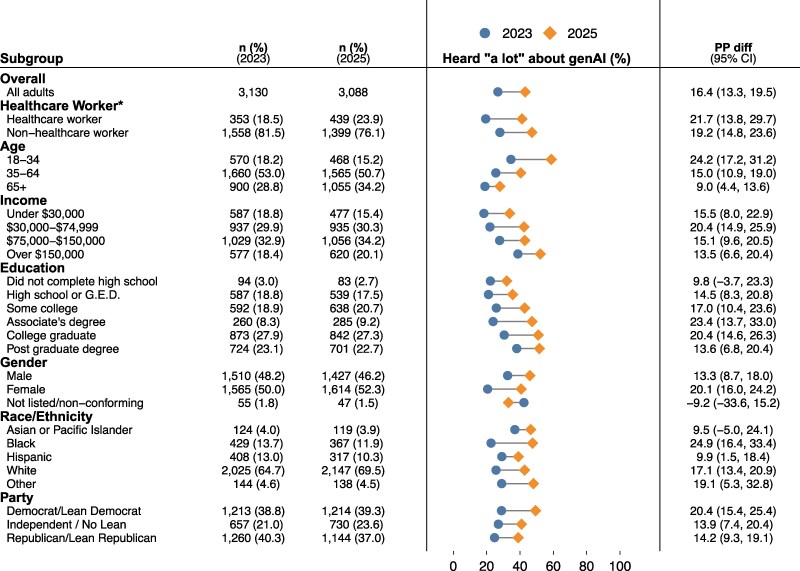
Study sample characteristics and awareness of generative AI among US adults, 2023 vs 2025. Unweighted sample characteristics and weighted percentage of survey respondents reporting they had heard “a lot” about generative AI among US adults surveyed on the Outbreaks Near Me participatory surveillance platform in May 2023 (N = 3130) and November 2025 (N = 3088), stratified by self-reported healthcare worker status, age, household income, education, gender, race/ethnicity, and political identity. Columns show unweighted sample sizes with group percentages, and percentage point (PP) differences between survey waves with 95% confidence intervals. Circles represent 2023 estimates and diamonds represent 2025 estimates. All estimates used inverse probability of selection weights targeting US Census distributions. *Healthcare worker status calculated only among those who self-report working full or part-time.

**Figure 2:**
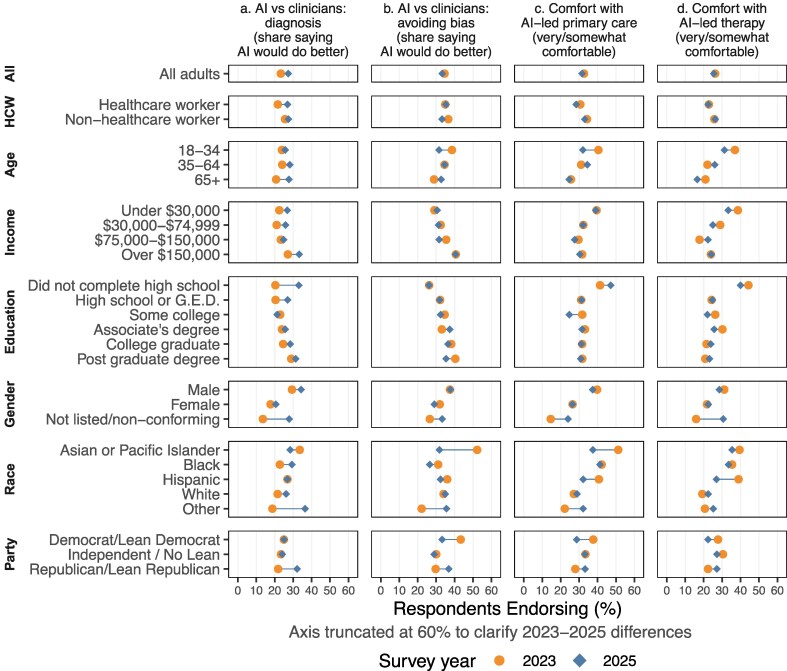
Perception of and comfort with generative AI in healthcare by demographic subgroup, 2023 vs 2025. Weighted percentage of survey responses to four generative AI perception measures among US adults surveyed on the Outbreaks Near Me participatory surveillance platform in May 2023 (N = 3130) and November 2025 (N = 3088) stratified by age, household income, education, gender, race/ethnicity, and political identity. Panels show (a) perception of AI as better than clinicians at diagnosis, (b) perception of AI as better at avoiding bias, (c) comfort with AI-led primary care (very/somewhat comfortable), and (d) comfort with AI-led therapy (very/somewhat comfortable). Circles represent 2023 estimates and diamonds represent 2025 estimates. All estimates used inverse probability of selection weights targeting US Census distributions.

Awareness of genAI among US adults rose (Figure 1), with 42.9% (95% confidence interval [95% CI]: 40.6, 45.3) reporting they had read or heard about genAI “a lot” in November 2025 [Total *N* = 3088] compared to 26.5% (95% CI: 24.5, 28.5) in May 2023 [Total *N* = 3130]. Overall comfort with AI-led primary care (−1.0 pp [95% CI: −4.2, 2.1]), AI-led therapy (−0.7 pp [95% CI: −3.7, 2.3]), and perceived bias superiority (−1.3 pp [95% CI: −4.4, 1.8]) all changed minimally between waves (Figure 2). Perceived diagnostic superiority rose slightly (4.0 pp [95% CI: 1.1, 6.9]).

Across demographic subgroups, awareness of genAI in healthcare increased unevenly (Figure 1) between 2023 and 2025, while perception changes varied in both magnitude and direction. We observed no change by gender or education, modest differences by healthcare worker status and income, and the largest differences by political identity, age, and race (Figure 2). Respondents who reported being a Democrat or leaning Democrat remained stable or became less likely to endorse AI as better at diagnostics (−0.1 pp [95% CI: −4.6, 4.7]), avoiding bias (−10.1 pp [95% CI: −15.2, −4.9]), comfort with AI-led primary care (−9.0 pp [95% CI: −14.1, −3.9]), and AI-led therapy (−5.4 pp [95% CI: −10.2, −0.6]). In contrast, respondents who reported being a Republican or leaning Republican showed the opposite pattern across the same measures (10.3 pp [95% CI: 5.5, 15.1], 7.1 pp [95% CI: 2.1, 12.1], 5.3 pp [95% CI: 0.3, 10.3], and 4.8 pp [95% CI: 0.0, 9.6], respectively). These trends flipped overall favorability, with Democrats more favorable in 2023 and Republicans in 2025.

GenAI perception patterns also differed by age, household income, and race (Figure 2). Adults aged 18–34 showed larger increases in awareness (24.2 pp [95% CI: 17.2, 31.2]) than those 65+ (9.0 pp [95% CI: 4.4, 13.6]), yet became less likely to endorse genAI as better at avoiding bias (−7.0 pp [95% CI: −13.9, −0.1]), compared to older adults (3.8 pp [95% CI: −1.5, 9.1]). Additionally, healthcare workers' perception of genAI's diagnostic capability was lower than non-healthcare workers in 2023, but the gap narrowed in 2025.

Between 2023 and 2025, Asian/Pacific Islander respondents became less favorable on AI as better at diagnosis (−5.2 pp [95% CI: −18.7, 8.4]), avoiding bias (−20.4 pp [95% CI: −34.6, −6.3]), and comfort with AI-led primary care (−13.8 pp [95% CI: −28.3, 0.7]), while White respondents trended positively on all three (Figure 2). Additionally, Hispanic respondents became less comfortable with AI-led therapy (−12.0 pp [95% CI: −20.4, −3.5]) and, to a lesser degree, AI-led primary care (−8.5 pp [95% CI: −17.1, 0.1]). Some differences were also seen by age and income, with younger adults (18–34) becoming less comfortable with AI-led primary care (−8.4 pp [95% CI: −15.4, −1.5]) and middle-income respondents ($75 000–$150 000) becoming more comfortable with AI-led therapy compared to households earning under $30 000.

In interaction models, the divergence between Democrats and Republicans was consistent, with interaction ORs ranging from 1.69 [95% CI: 1.19, 2.39] to 2.11 [95% CI: 1.54, 2.90] across the four measures (Table S2). Healthcare workers did not differ from non-healthcare workers on any of the four measures (AI better at diagnosis: OR 1.21 [95% CI: 0.76, 1.93]; AI better at avoiding bias: OR 1.18 [95% CI: 0.77, 1.81]; comfort with AI-led primary care: OR 0.95 [95% CI: 0.61, 1.48]; comfort with AI-led therapy: OR 0.96 [95% CI: 0.59, 1.56]). On genAI avoiding bias in healthcare, adults 65+ trended more favorably than those 18–34 (OR [95% CI]: 1.63 [1.10, 2.42]), while Asian or Pacific Islander respondents trended less favorably than White respondents (OR [95% CI]: 0.41 [0.22, 0.77]). Adults aged 35–64 likewise trended more favorably than those 18–34 on comfort with AI-led primary care (OR 1.68 [95% CI: 1.17, 2.41]) and AI-led therapy (OR 1.60 [95% CI: 1.10, 2.33]). Hispanic respondents also trended less favorably than White respondents on comfort with AI-led therapy (OR 0.48 [0.31, 0.75]) and primary care (OR 0.63 [0.42, 0.96]). Middle-income respondents (those earning $75 000 to $150 000) showed a more favorable trend than households earning under $30 000 on comfort with AI-led therapy (OR [95% CI]: 1.67 [1.05, 2.68]). In exploratory models including a survey year-by-political identity-by-healthcare worker status interaction, healthcare worker status did not modify the partisan divergence. The partisan divergence was also not attenuated after adjustment for urban-rural residence.

## Discussion

Despite the widespread enthusiasm and investment in genAI within the healthcare sector,^15^ overall positive sentiment among US adults has remained relatively stagnant, with little increase in comfort with (or preference for) AI-led care. The promise of genAI in healthcare depends as much on public trust as on technical capability, regulation, or incentives.^16^ The near doubling of awareness alongside flat comfort suggests trust is not organically growing alongside exposure and may continue to be a barrier to broader clinical AI adoption. While aggregate measures showed little movement, this stability masked meaningful trends across subgroups, including political leaning, age, race, and, to a lesser extent, income. Additionally, despite trailing in 2023, healthcare workers' sentiment towards AI's diagnostic capabilities aligned more closely with non-healthcare workers by 2025, perhaps reflecting growing professional awareness of genAI.

Self-identified Democrats and Republicans diverged sharply in their trends related to comfort with genAI in healthcare between 2023 and 2025, with Republicans becoming more favorable across all measures while Democrats declined. This pattern is consistent with broader partisan sorting around healthcare^17^ and AI^18^ trust, and it raises concerns that public receptivity to genAI in healthcare may become increasingly shaped by political identity rather than clinical evidence or personal experience. GenAI is often promoted as a mechanism to extend care where clinicians are scarce, including rural areas facing care shortages. Yet the partisan divergence we observe persisted after accounting for urban-rural residence, suggesting receptivity is driven by political identity more than need. As with vaccination, these early partisan differences could harden into an identity marker through a recursive loop between political cues and public attitudes, further politicizing AI-enabled care and exacerbating the disparities it could help address. With AI healthcare regulation actively taking shape,^7^ bipartisan engagement may be needed to prevent these divides from deepening.

The decline in AI favorability among Hispanic and Asian or Pacific Islander respondents is consistent with documented concerns about algorithmic bias in minority populations, though small subgroup sizes warrant caution. Additionally, middle-income individuals trended more favorably toward AI-led therapy while those with lower incomes trended inversely. If populations with traditionally less access to healthcare resources are growing less receptive to genAI, efforts to leverage AI tools to address equity gaps^19^ may be undermined. Notably, this gap between awareness and comfort was sharpest among younger adults. Despite showing the steepest rise in awareness, adults 18–34 became less comfortable with AI-led care, further supporting that awareness alone is not enough to slow this growing fracture in trust.

## Limitations

As a repeated cross-sectional design with independent samples, observed changes reflect population-level shifts rather than individual attitude changes, and causation cannot be inferred. The online nonprobability sampling approach may not fully represent the US population despite census-based weighting, and some subgroup estimates generated wide confidence intervals. Because participants were recruited through end-page river sampling and then opted into a health surveillance system, the sample likely overrepresents individuals with greater internet access and health engagement, though identical recruitment across waves limits the effect on observed changes. We also cannot rule out compositional change within subgroups across waves, as a subgroup may be differentially selected in each year, complicating interpretation of its trend; however, weights in each wave were calibrated to the same Census targets, and the partisan divergence appeared consistently across four independently measured outcomes. Additionally, respondents' understanding of genAI likely evolved, making it difficult to disentangle shifting opinions from shifting definitions.

## Conclusion

Sentiment toward genAI in healthcare has not kept pace with the growth in attention these tools have received. As AI integration accelerates, policymakers and health systems should recognize that public trust in these tools is polarizing along partisan and demographic lines, and that deployment without targeted trust-building risks widening rather than narrowing existing disparities.

## Supplementary Material

qxag148_Supplementary_Data
