# Supplementary material for: Trends in perception of generative AI in healthcare by US adult demographics and political identity
Source: Health Aff Sch. 2026 Jun 10;4(7):qxag148. doi: 10.1093/haschl/qxag148 (PMC13330703; doi:10.1093/haschl/qxag148)
Supplement: qxag148_Supplementary_Data [file qxag148_supplementary_data.zip › Supplement.docx]

**Supplementary Material**

Trends in Perception of Generative AI in Healthcare by US Adult Demographics and Political Identity

Rader, Gertz, Cantor, McBain & Brownstein

**Supplementary Table 1**. Weighted and unweighted Outbreaks Near Me survey sample demographics compared to the 2019-2023 5-year American Community Survey population estimates

|  |  | 2023 | | 2025 | |  |
| --- | --- | --- | --- | --- | --- | --- |
| Subgroup |  | Unweighted % | Weighted % | Unweighted % | Weighted % | ACS |
| Age | 18-34 | 18.2 | 29.4 | 15.2 | 29.2 | 29.3 |
| Age | 35-64 | 53.0 | 49.0 | 50.7 | 49.2 | 49.1 |
| Age | 65+ | 28.8 | 21.6 | 34.2 | 21.6 | 21.6 |
| Income | Under $30,000 | 18.8 | 18.4 | 15.4 | 18.4 | 18.4 |
| Income | $30,000-$74,999 | 29.9 | 29.4 | 30.3 | 29.5 | 29.6 |
| Income | $75,000-$150,000 | 32.9 | 30.3 | 34.2 | 30.2 | 30.1 |
| Income | Over $150,000 | 18.4 | 21.9 | 20.1 | 21.9 | 21.8 |
| Education | Did not complete high school | 3.0 | 10.4 | 2.7 | 10.7 | 10.6 |
| Education | High school or G.E.D. | 18.8 | 26.1 | 17.5 | 26.3 | 26.2 |
| Education | Some college | 18.9 | 19.5 | 20.7 | 19.3 | 19.4 |
| Education | Associate's degree | 8.3 | 8.8 | 9.2 | 8.8 | 8.8 |
| Education | College graduate | 27.9 | 21.4 | 27.3 | 21.2 | 21.3 |
| Education | Post graduate degree | 23.1 | 13.9 | 22.7 | 13.8 | 13.7 |
| Gender | Male | 48.2 | 48.1 | 46.2 | 48.4 | 49.0 |
| Gender | Female | 50.0 | 50.1 | 52.3 | 50.1 | 51.0 |
| Race/Ethnicity | White | 64.7 | 58.4 | 69.5 | 58.2 | 58.2 |
| Race/Ethnicity | Black | 13.7 | 12.1 | 11.9 | 12.0 | 12.0 |
| Race/Ethnicity | Hispanic | 13.0 | 18.8 | 10.3 | 18.9 | 19.0 |
| Race/Ethnicity | Asian or Pacific Islander | 4.0 | 5.9 | 3.9 | 5.9 | 5.9 |
| Race/Ethnicity | Other | 4.6 | 4.8 | 4.5 | 4.9 | 4.9 |

**Supplementary Table 2**. Interaction Odds Ratios from Separate Logistic Regression Models Assessing Differential Changes in Perception of Generative AI in Healthcare Between 2023 (N=3,130) and 2025 (N=3,088) by Demographic Subgroup

| **Subgroup** |  | **AI better at diagnosis** | **AI better at avoiding bias** | **Comfort with AI-led primary care** | **Comfort with AI-led therapy** |
| --- | --- | --- | --- | --- | --- |
| Healthcare Worker | Non-HCW (ref) | ref | | | |
|  | HCW | 1.21 (0.76, 1.93) | 1.18 (0.77, 1.81) | 0.95 (0.61, 1.48) | 0.96 (0.59, 1.56) |
| Age | 18 to 34 (ref) | ref | | | |
|  | 35 to 64 | 1.12 (0.75, 1.66) | 1.36 (0.95, 1.95) | 1.68 (1.17, 2.41)** | 1.60 (1.10, 2.33)* |
|  | 65+ | 1.33 (0.86, 2.05) | 1.63 (1.10, 2.42)* | 1.37 (0.91, 2.06) | 0.96 (0.62, 1.50) |
| Income | Under 30k (ref) | ref | | | |
|  | 30k-75k | 1.04 (0.63, 1.70) | 0.88 (0.56, 1.38) | 1.01 (0.65, 1.57) | 1.03 (0.65, 1.63) |
|  | 75k-150k | 0.86 (0.53, 1.40) | 0.78 (0.50, 1.22) | 0.92 (0.60, 1.43) | 1.67 (1.05, 2.68)* |
|  | Over 150k | 1.06 (0.64, 1.76) | 0.93 (0.58, 1.49) | 0.96 (0.60, 1.53) | 1.27 (0.77, 2.10) |
| Education | HS/GED (ref) | ref | | | |
|  | Less than HS | 1.34 (0.62, 2.91) | 1.01 (0.48, 2.15) | 1.25 (0.64, 2.46) | 0.82 (0.41, 1.63) |
|  | Some college | 0.63 (0.39, 1.01) | 0.92 (0.60, 1.40) | 0.70 (0.46, 1.07) | 0.77 (0.49, 1.23) |
|  | Associates | 0.77 (0.43, 1.36) | 1.23 (0.73, 2.07) | 0.92 (0.54, 1.56) | 0.78 (0.44, 1.38) |
|  | College grad | 0.85 (0.55, 1.31) | 0.94 (0.64, 1.39) | 0.97 (0.65, 1.44) | 1.11 (0.72, 1.72) |
|  | Post grad | 0.78 (0.50, 1.22) | 0.82 (0.54, 1.24) | 0.95 (0.62, 1.46) | 1.11 (0.69, 1.79) |
| Gender | Female (ref) | ref | | | |
|  | Male | 1.04 (0.76, 1.43) | 1.16 (0.88, 1.54) | 0.91 (0.68, 1.21) | 0.85 (0.61, 1.16) |
| Race/Ethnicity | White (ref) | ref | | | |
|  | Asian/PI | 0.61 (0.31, 1.18) | 0.41 (0.22, 0.77)** | 0.52 (0.28, 0.98)* | 0.69 (0.36, 1.33) |
|  | Black | 1.09 (0.69, 1.72) | 0.77 (0.50, 1.18) | 0.88 (0.58, 1.34) | 0.76 (0.48, 1.19) |
|  | Hispanic | 0.78 (0.49, 1.22) | 0.82 (0.54, 1.24) | 0.63 (0.42, 0.96)* | 0.48 (0.31, 0.75)** |
| Political Party | Democrat (ref) | ref | | | |
|  | Independent | 1.03 (0.69, 1.55) | 1.45 (1.00, 2.10)* | 1.48 (1.02, 2.16)* | 1.14 (0.76, 1.71) |
|  | Republican | 1.69 (1.19, 2.39)** | 2.11 (1.54, 2.90)*** | 1.93 (1.39, 2.68)*** | 1.73 (1.20, 2.50)** |

* p < 0.05; ** p < 0.01; *** p < 0.001; Asterisks shown for reference - interpretation emphasizes magnitude and precision of estimates in accordance with American Statistical Association recommendations.
